# Multiparametric analysis of host and parasite elements in new world tegumentary leishmaniasis

**DOI:** 10.3389/fcimb.2022.956112

**Published:** 2022-08-09

**Authors:** Bruna Caroline de Carvalho, Tamires Vital, Jaqueline Osiro, Ciro Martins Gomes, Elza Noronha, Bruno Dallago, Ana de Cássia Rosa, Juliana Lott Carvalho, Luciana Hagström, Mariana Hecht, Nadjar Nitz

**Affiliations:** ^1^ Interdisciplinary Laboratory of Biosciences, Faculty of Medicine, University of Brasília, Brasília, Brazil; ^2^ Dermatology Diagnostic Group, Dermatomicology Laboratory, Faculty of Medicine, University of Brasília, Brasília, Brazil; ^3^ Brasília University Hospital, University of Brasília, Brasília, Brazil; ^4^ Laboratory of Animal Welfare, Faculty of Agronomy and Veterinary medicine, University of Brasília, Brasília, Brazil

**Keywords:** tegumentary leishmaniasis, symptoms, host, parasite, pathophysiology, correlation analysis.

## Abstract

Tegumentary leishmaniasis is a tropical disease caused by protozoa of the genus *Leishmania.* Clinically, the disease presents a broad spectrum of symptoms, the mechanisms underlying the development of lesions remaining to be fully elucidated. In the present work, we performed a correlation and multiparametric analysis to evaluate how parasite- and host-related aspects associate with each other, and with the different clinical manifestations of tegumentary leishmaniasis. This cross-sectional study involved 75 individuals from endemic areas of Brazil, grouped according to their symptoms. *Leishmania* species were determined by DNA sequencing, and parasite load, antibody production, and cytokine profile were evaluated by kDNA qPCR, ELISA, and flow cytometry. Data were analyzed using the Chi-square test, principal component analysis, canonical discriminant analysis, and correlation analysis. Among the recruited patients, 23 (31%) were asymptomatic, 34 (45%) had primary cutaneous leishmaniasis, 10 (13%) presented recurrent cutaneous leishmaniasis, and eight (11%) had mucocutaneous leishmaniasis. *Leishmania* species identified included *L. amazonensis, L. braziliensis*, and *L. guyanensis*. Surprisingly, no *Leishmania* RNA virus infection was detected in any sample. In summary, our work showed that parasite load, antibody production, and cytokine levels alone are not determinants for tegumentary leishmaniasis symptoms. However, the correlation analysis allowed us to observe how these factors are correlated to each other within the groups, which revealed a unique network for each clinical manifestation. Our work reinforces the complexity of tegumentary leishmaniasis outcomes - which are associated with multiple host and parasite-related elements and provides a holistic model of the disease.

## Introduction

Tegumentary leishmaniasis (TL) is a vector-borne neglected tropical disease caused by species of the genus *Leishmania*. TL occurs in 92 countries, and it is the sixth most prevalent dermatological disease in the world (Gosch et al., 2017; World Health Organization, 2022).

TL shows a broad spectrum of clinical manifestations: cutaneous leishmaniasis (CL), disseminated leishmaniasis (DL), diffuse cutaneous leishmaniasis (DCL), and mucocutaneous leishmaniasis (MCL) (Campos et al., 2018). CL is the most common and least severe clinical form of TL, being characterized by the formation of painless ulcers with rounded shape and defined elevated borders. DL is identified by the presence of papular lesions, and it is known to occur simultaneously or subsequently to CL. Finally, DCL is characterized by parasite spread, with ulcers covering the entire body, and MCL is diagnosed by the identification of oral and nasal mucosa lesions (Marra et al., 2014; Fernández-Figueroa et al., 2016; Guimarães et al., 2016; Vernal et al., 2016).

The symptoms observed in TL are of complex etiology and the mechanisms involved in the development of lesions are not completely elucidated (Dunning, 2009). At this point, it is known that several factors seem to modulate the leishmaniasis pathogenesis, such as *Leishmania* strain, parasite load, and even the presence of the *Leishmania* RNA virus (LRV1) (Ives et al., 2011; Kuhlmann et al., 2017) Besides, the host immune system characteristics and its capacity to eliminate the parasite is another major feature that contributes to the comprehension of why TL ranges from an asymptomatic to a severe disease (Gupta et al., 2013; Pace, 2014).

Several works have been carried out to investigate the role of specific clinical parameters over TL outcomes. Nevertheless, most studies fail to assess concurrent variables and don’t use multiparametric analysis. In recent years, the complex and multifactorial nature of TL pathophysiology has become increasingly clear, underscoring the need for experimental and statistical approaches that enable the investigation of how multiple aspects of the disease influence each other and how all variables taken together explain the broad spectrum of clinical manifestations observed in TL.

The present study aims to shed light into the TL pathophysiology, investigating how different parasite (i.e., *Leishmania* species, parasite load, and LRV1 presence), and host elements (i.e., circulating cytokine and antibody levels) correlate with each other and how this correlation is associated to the severity of the symptoms which is represented by different clinical manifestations.

## Methods

### Study population

The study population consisted of 30 patients from Corte de Pedra (Bahia, Brazil), and 45 participants treated at the Dermatology Ambulatory of the University Hospital of Brasília (UHB) (Brasília, Federal District., Brazil). All patients lived in TL endemic areas. Blood samples were collected from all patients and biopsies were collected from 51 individuals showing lesions during the medical appointment. Patients were classified as TL positive if serological or molecular tests were positive, and grouped into four categories, according to clinical manifestations: (1) *asymptomatic*, patients with positive qPCR or serology test, no detectable lesions, but who had already presented lesions in the past; (2) *primary CL*, individuals whose first lesions were detected at the time of recruitment; (3) *recurrent CL*, defined as patients who developed TL after previous adequate treatment and lesion healing, and; (4) *MCL*, patients with lesions in mucosal areas. HIV+ individuals, patients younger than 18 years, and indigenous subjects were excluded.

### Montenegro skin test

The Montenegro skin test was performed on patients from Corte de Pedra as part of the routine of the local research group. Nevertheless, the results were not considered for TL diagnosis. Briefly, the test was executed using the *Leishmania* antigen prepared in the Laboratory of Immunology of the University Hospital of Salvador, Salvador, Bahia, Brazil, using *L. amazonensis (*strain MHOM/BR/86/BA125) promastigotes, at a concentration of 5x10^6^ promastigotes per ml, equivalent to 250 µg ml^−1^ antigen. An induration of ≥5 mm was considered positive (Reed et al., 1986).

### Sample collection

Blood samples were collected in tubes containing either EDTA or clot activator and separator gel to obtain serum. Skin biopsies were taken from the borders of active lesions, using a 4-mm diameter punch, after the application of local anesthetic. Blood, serum and biopsy samples were immediately stored in RNA*later* Stabilization Solution (Thermo Fisher Scientific, USA) for posterior RNA and DNA extraction, ELISA, and cytokine quantification.

### DNA extraction

DNA was extracted from blood and skin biopsies using the *Biopur Mini Spin Plus* Kit (Biometrix, Brazil). Samples were quantified in a NanoVue Plus (GE Healthcare, UK) and DNA quality was evaluated by PCR targeting the *β-actin* gene using the following primers: 5’- ATC TGG CAC CAC ACC TTC TAC AAT GAG CTG CG-3’ and 5’-CGT CAT ACT CCT GCT TGC TGA TCC ACA TCT GC-3’.

### Viral RNA extraction, cDNA synthesis and LRV1 detection

Viral RNA was extracted from blood and skin biopsies using the *Biopur Mini Spin Virus RNA* Kit (Biometrix, Brazil). The resultant viral RNA samples were quantified in a NanoDrop (Thermo Fisher Scientific, USA). cDNA synthesis was performed using the SuperScript ™ IV First-Strand cDNA Synthesis Reaction Kit (Invitrogen, USA), and quantitative reverse transcription PCR (RT-qPCR) was performed using the following primers for the ORF1 region of the LRV1 genome: 5’- ATG CCT AAG AGT TTG GAT TCG -3’ and 5’ ACA ACC AGA CGA TTG CTG TG - 3’. The reactions were performed in a final volume of 20 µL, consisting of 1x Universal SYBR Green PCR master mix (Applied Biosystems, USA), 10 µM of each primer and 2 μL of cDNA. Reaction conditions were as follows: 95°C for 10 minutes and 40 cycles at 94°C for 15 s, 53°C for 40 s and 72°C for 10 s. The melting curve was standardized with denaturation of 95°C for 15 s, followed by 60°C for 60 s and 95°C for 15 s. The reactions were carried out in an Applied Biosystems™ QuantStudio 3 (Thermo Fischer Scientific, USA). *L. guyanensis* LRV1^+^ (MHOM/BR/1989/IM35) cDNA was used as positive control, and *L. infantum* (MCER/BR/79/M6445) cDNA was used as negative control sample in all assays. The PCR reaction mixture containing water in place of template (blank) was also used as contamination control in each assay.

### Quantitative PCR to determine parasite load

Quantitative PCR (qPCR) was performed using primers to *Leishmania* kDNA minicircle to determine parasite load. The primer sequences were as follows: 5’-GGC CCA CTA TAT TAC ACC AAC CCC-3’ and 5’-GGG GTA GGG GCG TTC TGC GAA-3’. The reactions were performed in a final volume of 20 µL, consisting of 1x Universal SYBR Green PCR master mix (Applied Biosystems, USA), 0.2 µM of each primer and 50 ng of genomic DNA. The reaction conditions were 94°C for 12 minutes, followed by 40 cycles of 94°C for 30 s, 55°C for 30 s and 72°C for 30 s. The melting curve was processed with 95°C for 5 s, followed by 50°C for 15 s and 95°C for 5 s. All qPCR assays included positive (*L. braziliensis* DNA) (MHOM/BR/2000/LTCP13396) and negative controls (blank and non-infected HEK cell DNA). To determine the number of parasites, a standard curve was built using serial dilution of *L. braziliensis* DNA (5 x 10^2^ to 5 x 10^-3^ ng/µL). The reactions were carried out in an Applied Biosystems™ QuantStudio 3 (Thermo Fischer Scientific, USA). The parasite load was calculated as equivalent parasites per 50 ng of DNA.

### Serological diagnosis

Anti-*Leishmania* IgG was detected in serum samples by ELISA. The assays were run in microplate wells sensitized with specific parasite antigens (0.1 μg/well of *L. braziliensis* soluble antigen) and incubated overnight in a humidified atmosphere at 37°C. Then, 150 μL of 1X Milk-PBS (pH 7.4, 5% w.v. skim milk) was added. Serum samples were diluted 1:100 using 1X Milk-PBS (pH 7.4, 2% w.v. skim milk) and added to the wells. After 2 h incubation at 37 °C in a humid chamber, the plates were washed and 50 μL/well of the secondary antibody was added. A second incubation was then performed with 50 μL of 1:500 dilution of peroxidase-conjugated anti-human IgG (Sigma-Aldrich, USA). Each well then received 50 μl/well of revealing solution (pnPP-p-nitrophenol phosphate - diluted in Diethanolamine buffer pH 9.8) and, after a 12-minute incubation in the dark, absorbance reading was performed at 490 nm using a BioTeK^®^-Synergy HT spectrophotometer. The following controls were used in all assays: two sera known to be positive for anti-*Leishmania* IgG, and ten sera from healthy individuals as negative controls. The cut-off values were determined using the mean value of the negative controls plus three standard deviations (Sarkari et al., 2014).

### Measurement of cytokine production

The cytokine profile Th1/Th2/Th17 (IL-2, IL-4, IL-6, IL-10, TNFα, IFN-y and IL-17A) of serum samples obtained from all 75 patients was determined using the CBA Human Th1/Th2/Th17 kit (BD Biosciences,USA). Briefly, the reconstituted cytokine standards and the thawed serum samples were processed using the LSRFortessa™ BD cytometer, according to the manufacturer’s instructions. Three hundred events or more were acquired for each cytokine bead. Data were analyzed using the FCAP software 3.0 (BD Biosciences, USA).

### Identification of *Leishmania* species

Conventional PCR (cPCR) was performed with LITS.R and L5.8S primers: 5’- TGA TAC CAC TTA TCG CAC TT-3’ and 5’- AAG TGC GAT AAG TGG TA-3’. The conditions were: 20 ng of DNA sample, 1x reaction buffer (20 mM Tris-HCl pH 8.4 and 50 mM KCl), 1 mM MgCl_2_, 0.25 μM of each primer, 0.2 mM dNTPs and 2.5 units of Taq DNA Polymerase (Invitrogen, USA) in a final volume of 25 µL. All amplifications were carried out on a T-100 Thermal Cycler thermocycler (Bio-Rad, USA) with an initial denaturation step at 95° C for 5 minutes, followed by 35 cycles of 95°C for 30 s, 58°C for 30 s, 72°C for 30 s and a final extension step of 72°C for 5 minutes. *Leishmania* kDNA minicircle primers were not recommended for the identification of species, although it is the most sensitive target, the amplified sequence does not allow the differentiation of *Leishmania* species (Conter et al., 2019). The amplification of *Leishmania* ITS1 region was performed in all patients which tested positive in the kDNA qPCR, amplicons could only be obtained in 19 patient samples. The positive PCR products were purified using Illustra GFX PCR DNA and gel band purification kit (GE Healthcare, USA) and sequenced at the Genomic Molecular Engineering Institute (São Paulo, Brazil). Sequences were edited using Geneious software (www.geneious.com) and analyzed using BLASTn algorithm (www.ncbi.nlm.nih.gov/BLAST).

### Statistical analysis

Qualitative dependent variables were submitted to the Chi-square test. Quantitative data were submitted to Shapiro-Wilk analysis and then, an one way ANOVA using Kruskal-Wallis test (PROC NPAR1WAY) was performed in order to verify differences in cytokines concentration between clinical manifestations. Pearson correlation analysis using PROC CORR, Principal Component Analysis using PROC PRINCOMP and canonical discriminant analysis using PROC CANDISC were used to evaluate quantitative data. All statistical analyzes were performed using the SAS^®^ program (v9.4, Cary, North Carolina). Statistical significance was assigned to p ≤ 0.05. Effect size achieved 0.39 and was calculated using GPower^®^ software (v3.1.9.4, 2019) and the following input parameters: 0.05 for α, 0.8 for power, 4 groups and sample size of 75 individuals. Hedges’g value was calculated using effect size calculator for T-Test on Social Science Statistics website (https://www.socscistatistics.com/effectsize/default3.aspx) and and obtained a mean Hedges’g value of 0.36.

## Results

From the 75 patients investigated, 23 (31%) were classified as asymptomatic, 34 (45%) as primary CL, 10 (13%) as recurrent CL and 8 (11%) as MCL. There was no patient with MCL in Corte de Pedra and no asymptomatic individual was recruited at the UHB (**Supplementary Material- Figure S1**). Most patients were male (63%) and were aged 30 to 59 years old (59%). Sex and age presented no statistically significant association with clinical symptoms (**Table 1**).

**Table 1:** Distribution of patients by age, sex, and clinical manifestations.

	Asymptomatic	Primary CL	Recurrent CL	MCL	p-value
**Gender**					0.154
**Male n, (%)**	10 (21)	25 (53)	7 (15)	5 (11)	
**Female n, (%)**	13 (46)	9 (32)	3 (11)	3 (11)	
**Age group**					0.156
**18 to 29 years n, (%)**	1 (9)	6 (54)	1 (9)	3 (28)	
**30 to 59 years n, (%)**	13 (28)	23 (52)	6 (15)	2 (5)	
**≥ 60 years n, (%)**	9 (43)	5 (29)	3 (14)	3 (14)	

CL, Cutaneous leishmaniasis; MCL, Mucocutaneous leishmaniasis.

**Figure 1:**
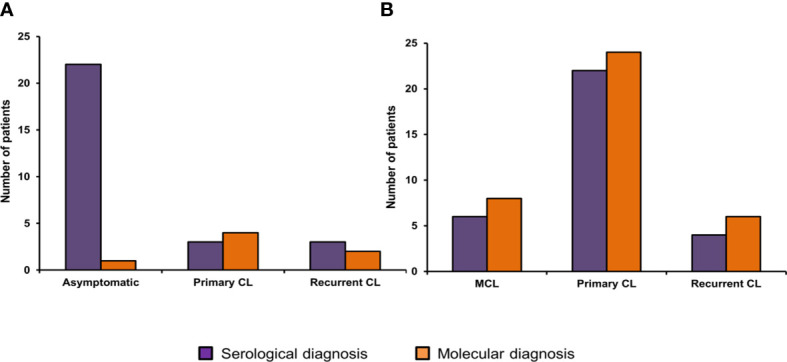
Clinical manifestation distribution of patients who tested positive for TL using serological (ELISA) and molecular diagnosis (kDNA qPCR). **(A)** Patients of Corte de Pedra. **(B)** Patients of the UHB. Legend: CL, Cutaneous leishmaniasis, MCL, Mucocutaneous leishmaniasis.

*Leishmania* kDNA qPCR standard curve parameters were R^2 =^ 0.970, slope= -3.228 and efficiency=104%, (**Supplementary Material- Figure S2**). Positive qPCR results were obtained in 45 (60%) patients for at least one of the samples (blood or skin biopsy). The ELISA diagnosis identified 60 positive patients, the sensitivity and specificity of the test being 80% and 100%, respectively. Molecular and serological tests agreed in 30 individuals (40%). There was no statistically significant difference between the diagnostic tests and the clinical groups, except for the asymptomatic group, in which ELISA has shown to be more sensitive (**Figure 1**).

The ELISA results revealed a generally very low antibody levels and there was no significant difference in antibody titers among the clinical groups, despite a higher production being observed in patients with recurrent CL (1.58 ± 0.50 U/µL) and with MCL (1.96 ± 0.46 U/µL), when compared to asymptomatic (0.99 ± 0.30 U/µL) and primary CL patients (1.09 ± 0.20 U/µL).

**Figure 2:**
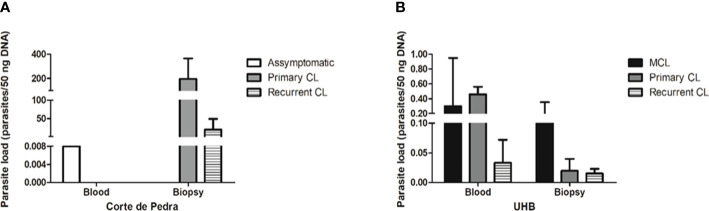
Parasite load of different samples of the studied groups, as determined by qPCR. **(A)** Parasite load (parasites/50 ng DNA) by sample type and clinical manifestation from patients of Corte de Pedra. **(B)** Parasite load (parasites/50 ng DNA) by sample type and clinical manifestation of patients attended at Brasília University Hospital (UHB). Legend: CL, Cutaneous leishmaniasis; MCL, Mucocutaneous leishmaniasis.

The correlation of clinical manifestations with parasitemia showed a different profile in the different patient cohorts. In Corte de Pedra only one blood sample, which was obtained from one asymptomatic individual, tested positive for qPCR. On the other hand, all patients with active lesions had positive results for skin biopsy qPCR for parasite detection. Surprisingly, though, the same patients did not show positive results for qPCR parasite detection in the blood. Considering the single blood sample that tested positive for qPCR, parasite load was estimated to be of 0.008 parasite equivalents per 50 ng of DNA (par. eq./50 ng of DNA). In skin biopsies, the mean number of par. eq./50 ng of DNA was of 196.4 for patients with primary CL, and 20.0 for individuals with recurrent CL (**Figure 2A**).

Considering the UHB patient cohort, the mean number of par. eq./50 ng of DNA in blood samples was of 0.30 for MCL patients, 0.46 for primary CL patients and 0.03 for recurrent CL patients. In the case of skin lesion biopsies, the mean number of par. eq./50 ng of DNA was of 0.164 for MCL patients, 0.02 for primary CL patients and 0.01 for recurrent CL patients (**Figure 2B**). Therefore, individuals from Corte de Pedra who had active lesions showed parasite loads approximately 1000 times greater than patients with active lesions recruited at UHB. Regardless of the observed discrepancies, parasitemia failed to significantly correlate with the severity of symptoms.

The *Leishmania* species could be successfully identified in 19 out of the 45 patients who tested positive for kDNA qPCR. *L. amazonensis* was identified in five individuals, all with primary CL. *L. braziliensis* was detected in eight individuals, five of which were classified as primary CL, two as MCL, and one as recurrent CL. *L. guyanensis* was identified in six individuals, four of which had primary CL and two of which presented recurrent CL. Of interest, the only species identified in samples obtained from the patients of Corte de Pedra was *L. braziliensis*, different from the samples obtained from UHB patients, which allowed the detection of the three *Leishmania* species (GenBank accession number MW865073 to MW865090) (**Supplementary material- Table S1**).

Since the LRV1 incidence for these two populations had never been investigated, one of the aims of the present work was also to verify the presence of the virus among the patients from the investigated cohorts, in order to assess any possible association with the severity of clinical manifestations. However, no sample presented LRV1 infection (**Supplementary Material- Figure S3**).

To verify the immune response of the studied population, the Th1/Th2/Th17 cytokine profile was determined in all serum samples. The statistical analysis showed that the cytokine levels when observed alone are not significant for the determination of the clinical manifestation (**Supplementary material- Table S2**).

**Figure 3:**
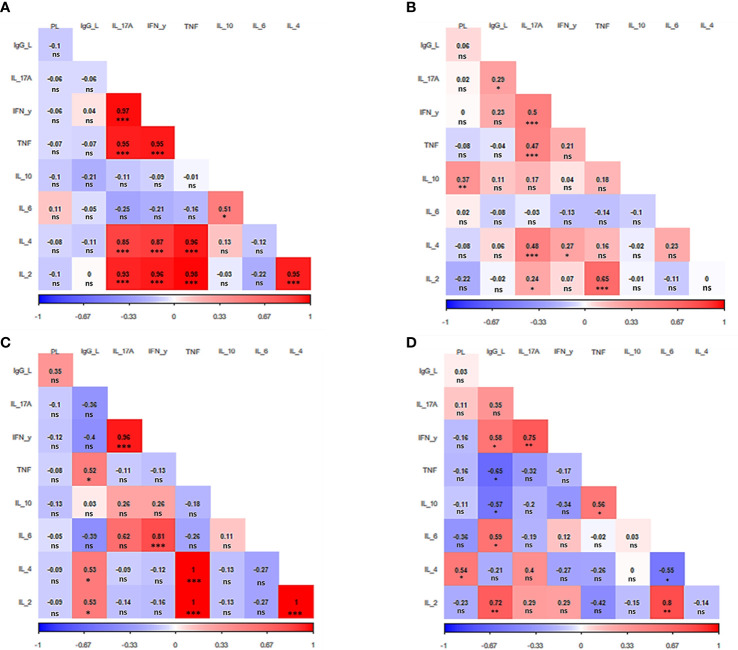
Correlation analysis heat map. The heat map shows the correlation among IgG anti-*Leishmania* titers, parasite load, and cytokine production for each clinical manifestation evaluated **(A)** Asymptomatic. **(B)** Primary cutaneous leishmaniasis. **(C)** Recurrent leishmaniasis. **(D)** Mucocutaneous leishmaniasis. PL. parasite load, IgG_L: anti-*Leishmania* titers IL: interleukin. IFN- γ: interferon γ. TNF: tumor necrosis factor. The value observed in each square is the Pearson correlation coefficient.*p < 0.05, **p < 0.01, ***p < 0.001, ns – non-significant.

Next, a correlation analysis was performed considering parasite load, antibody titers and cytokine levels within the groups. This correlation analysis allowed us to determine how these parameters are associated to each other and how they contribute with the pathophysiology of TL. Such an analysis revealed a different correlation pattern among factors for each clinical manifestation (**Figure 3**).

In the case of asymptomatic patients, five cytokines stood out, which were directly and strongly related to each other. They are IL-2, IL-4, IL-17A, IFN-y, and TNF (p<0.001) (**Figure 3A**). The same group of cytokines also attracts attention in patients with primary CL. Although not all these cytokines are related to each other, once again it is observed the direct correlation between all of them i.e., IL-2 (p<0.05), IL-4, IFN-y, and TNF (p<0.001) with IL17-A. In addition, as in asymptomatic patients, IL-2 and TNF are also strongly correlated (p<0.001). The primary CL group also presented a direct relation between parasite load and the releasing of IL-10 (p<0.05) (**Figure 3B**).

Patients with recurrent leishmaniasis presented a positive correlation between the levels of IL-2, IL-4 and TNF (p<0.001). Moreover, IFN-y was also directly correlated with IL-6 and IL-17A (p<0.001). The antibody titers had a greater effect on cytokine levels in patients from this group, presenting a positive correlation with IL-2, IL-4, and TNF (p<0.05) (**Figure 3C**).

Regarding the group of MCL patients, it was observed a direct relation between IL-2 and IL-6 (p<0.01), while IL-6 was inversely related to IL-4 (p<0.05). It was also verified a positive correlation between TNF and IL-10 (p<0.05). As seen in all other clinical manifestations, once again IFN-y was directly related to IL17-A (p<0.01). The parasite load was correlated with the increase of IL-4 p<0.05). Of interest, the release of TNF and IL-10 were inversely associated with the IgG production (p<0.05),while a direct relation was observed for IL-2 (p<0.01), IL-6, and IFN-y (p<0.001) (**Figure 3D**).

To gain a better insight into these parameters, we performed Principal Component Analysis (PCA). The first two eigenvectors of PCA explained 45.8% of variation observed and showed a collaborative association between IL-17A and IFN-y as well as between IL-6, IL-10 and parasite load and, in another pictorial cluster, IL-4, IL-2 and TNF (**Figure S4**).

**Figure 4:**
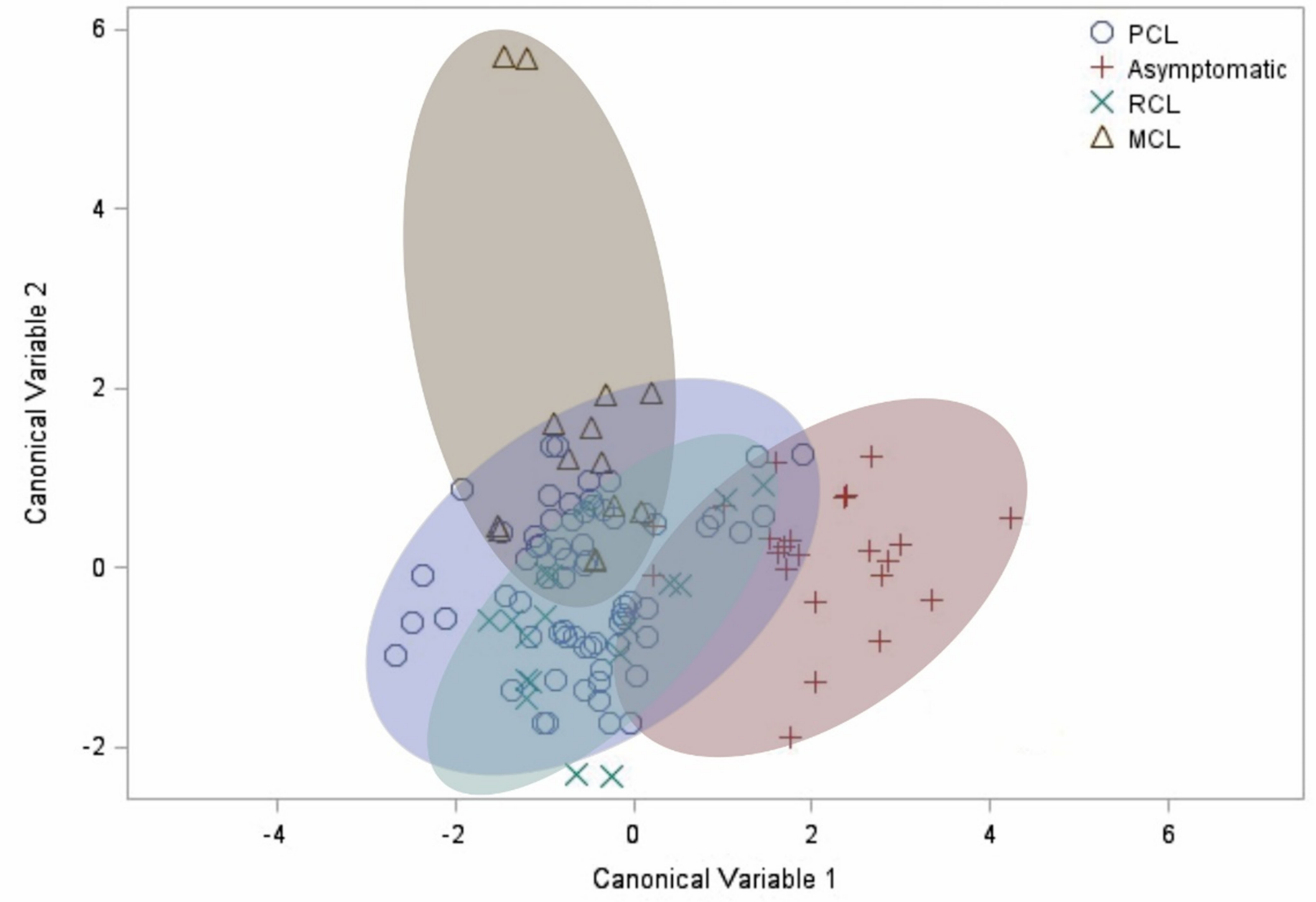
Canonical discriminant analysis. Each symbol represents a patient. The shaded clusters are dispersion ellipses for the four clinical manifestations. PCL, primary cutaneous leishmaniasis. RCL, recurrent cutaneous leishmaniasis. MCL, mucocutaneous leishmaniasis.

Finally, after observing the differences in how the evaluated factors correlate differently for each clinical manifestation, the canonical discriminant analysis was carried out. It demonstrated a clear distinction between the cluster of asymptomatic patients compared to the other groups (**Figure 4**). This result confirms the profile differences previously observed in the correlation analysis, in which the asymptomatic group showed a unique pattern.

## Discussion

Despite all the acquired knowledge regarding different elements of TL pathogenesis, there is still no consensus on how parasite- and host-related elements may connect to each other and influence clinical outcome. In this study, we evaluated the influence of several aspects, investigating how parasite-related factors (parasite load, species diversity and the presence of LRV1), as well as host-related factors (gender, age, place of residence and immune response) might contribute to the clinical manifestations of TL embodied here in four categories (asymptomatic, primary CL, recurrent CL, and MCL). These categories were used for all analysis and does not properly mean “disease severity” as for example, a patient could show a lot of skin lesions in any different severity ranges and does not show MCL.

The largest number of recruited patients were men aged between 30 and 59 years. Such observation was expected since middle-aged men are usually involved in agricultural activities, close to areas with the presence of the vector. Besides, physiological factors such as hormonal differences may also contribute to this finding, as previously published (Diniz et al., 2012; Guerra-Silveira and Abad-Franch, 2013).

An elevated parasitemia was detected in skin lesion biopsies obtained from Corte de Pedra patients, which may be related to genetic characteristics of the local *L. braziliensis.* Nevertheless, in our hands, parasite burden could not be associated with clinical manifestations of TL, as reported by Pereira et al. (de Pereira et al., 2017). In symptomatic patients from UHB, the parasite load of blood and biopsies did not present significant statistical differences. However, in Corte de Pedra patients, the symptomatic individuals with high parasite load on skin lesions had undetectable parasitemia in the blood, confirming that skin lesion biopsies are more suitable than blood for the diagnosis of TL (De Almeida et al., 2017). The tropism for the lesioned region may also be explained by possible characteristics of the Corte de Pedra *L. braziliensis* strain. Importantly, asymptomatic patients showed undetectable parasite loads, a determinant factor to explain the higher sensitivity of serological tests compared to molecular ones (Jara et al., 2013).

Some studies with visceral leishmaniasis indicate that there is a strong correlation between disease progression and the increase in antibody titers (Hasker et al., 2014; Singh et al., 2014). Nevertheless, in the present work, no significant differences in antibody production could be detected considering the clinical groups studied. Therefore, such a parameter cannot be significantly associated with higher chances of a certain clinical manifestation, despite the higher antibody titers detected in patients with MCL or recurrent CL, compared to the asymptomatic and primary CL patients.

The *Leishmania* species was identified in 19 patient samples. The low number of samples in which the parasite species could be identified compared to the number of samples that tested positive using molecular assays can be explained, at least in part, by the difference between the primers used in each assay. While the *Leishmania* kDNA minicircle primers present high sensitivity for *Leishmania* diagnosis, mainly due to the higher number of copies of the minicircle per cell, the *Leishmania* ITS1 region primers are widely used for identification of the species (Bensoussan et al., 2006; Galluzzi et al., 2018; Conter et al., 2019). In addition, the low parasitemia of most samples was also a limitation for sequencing.

This work reinforces that the parasite strains may be determinant to the clinical outcome of TL (Handler et al., 2015). The circulation of *L. amazonensis* and *L. braziliensis* has already been described in the studied areas (de Pereira et al., 2013; Souza Castro et al., 2018), and it is important to emphasize that, in the case of *L. braziliensis*, this was the only *Leishmania* species found in the area of Corte de Pedra, similar to the observations of Silva et al. (Silva et al., 2017). This species was also the only one detected in MCL patients, also corroborating previous works that indicate *L. braziliensis* as the *Leishmania* species more frequently associated with MCL cases in Brazil.

Regarding *L. guyanensis*, this is the first time that this species was reported in the Federal District. Still, this result must be interpreted with caution, given that the Federal District is a place of constant migration. Besides, during medical treatment, patients tend to inform the address of local relatives, since the centralized health system only allows the medical appointment of local residents. Therefore, it is difficult to confirm autochthonous cases. Finally, *L. guyanensis* was also the species that caused the most part of recurrent CL, confirming previous reports of *L. guyanensis* therapeutic resistance (Goto and Lindoso, 2010).

Although LRV contributes to ATL severity (de Carvalho et al., 2019), the lack of LRV1 in our samples suggests that RNA virus is not a determinant factor for disease progression and for drug resistance, corroborating prior studies (de Pereira et al., 2013; Ito et al., 2015; Valencia et al., 2022). The fact that the presence of this virus seems to be geographically limited to the northern region of Brazil also contributes to the comprehension of our failure to detect LRV in the investigated samples (de Pereira et al., 2013).

The investigation of immune response elements such as cytokine levels greatly contributes to disease comprehension. Our results showed the correlation of pro-inflammatory cytokines, especially IFN-γ, was predominant in all groups, independently of the clinical status (Maspi et al., 2016). This was more evident in the case of asymptomatic patients, which corroborates the results found by Ibarra-Meneses et al., 2022., and confirms that IFN-γ release is critical to *Leishmania* clearance and is associated with host protection against TL. Furthermore, IFN-γ response has already been observed in asymptomatic patients from endemic areas in Peru, which was associated with the exposure time to *Leishmania* (Best et al., 2018). The same pattern is observed in the present work since the asymptomatic patients are also from endemic areas for TL.

Likewise, the asymptomatic patients had a remarkable correlation for circulating levels of most Th1, Th2, and Th17 cytokines analyzed compared to the other TL patients, suggesting an immunological balance between inflammatory and modulatory immune events, leading to better control of the disease in this group. In turn, the Th1/Th2/Th17 imbalance in patients with different clinical forms of CL is a complex issue and some studies have associated the enhancement of specific cytokines with the immunopathology of TL (Carvalho et al., 2007; Dembic, 2015; Osero et al., 2020).

The correlation between IL-17A with pro-inflammatory and anti-inflammatory cytokines in all clinical groups attests to the double role of IL-17A, which can be associated with protective effects or with the enhancement of the disease severity (Banerjee et al., 2016). Of interest, we detected a positive correlation between IL-17A and IFN-γ production in all the groups analyzed. Although it has been described that IFN-γ suppresses the differentiation of Th17 lymphocytes, we observed that there is a synergistic pro-inflammatory action of these cytokines, that may contribute to the immunopathology of the TL (Newcomb et al., 2009; Oliveira et al., 2014; Maspi et al., 2016).

Apart from the relation between IL17-A and IFN-γ, patients with primary CL also showed a correlation of IL-17A with IL-2, IL-4, and TNF. These cytokines are often described in Th1 and Th2 responses and they can be considered bifunctional cytokines, for this reason, they are not a good marker for predicting clinical manifestation. In this sense, IL-2 may contribute to susceptibility or resistance in leishmaniasis, promoting activation of Th1 response as well as TNF which explains the strong correlation between these cytokines (Maspi et al., 2016). The positive relation between IL-2 and TNF was also observed in asymptomatic and recurrent CL patients. The correlation analysis also indicated that parasite load positively correlates with high levels of IL-10 in primary CL patients, corroborating previous works with visceral leishmaniasis, in which parasite survival could be benefited by IL-10 synthesis (Verma et al., 2010; Bhattacharya et al., 2016).

Patients with recurrent CL presented the cytokines IL-2, IL-4, and TNF correlated with each other. The relation between IL-2 and TNF has already been elucidated, however, IL-2 also can stimulate the proliferation of Th2 cells through the production of IL-4 and the association between these cytokines had already been demonstrated in BALB/c mice infected with L. major (Heinzel et al., 1993; Maspi et al., 2016). The correlation between TNF and IL-4 was surprising since TNF mediates Th1 response. Traditionally, IL-4 is related to an anti-inflammatory response, but evidence suggests that IL-4 is implicated to play a major paradoxical role in *Leishmania* infections. Previous studies demonstrated that IL-4 can instruct Th1 anti-leishmanial responses, but the protective effects of IL-4 are still under investigation (Biedermann et al., 2001; Hurdayal and Brombacher, 2014).

Another controversial cytokine is IL-6. Studies suggest that IL-6 may influence both pro-inflammatory/anti-inflammatory responses. Our results demonstrated an interesting association between IL-6 and IFN-γ release in the recurrent CL group. In this sense, Murray (2008) verified that IL-6 depleted mice enhanced control of *L. donovani* replication with increased levels of circulating IFN-γ (Murray, 2008).

MCL patients presented the most complex profile of correlation among the factors evaluated. It is clear the double role that IL-6 plays in this clinical manifestation. Unexpectedly, MCL patients presented a direct correlation between Il-6 and IL-2 levels, which is not a common find, since this cytokine contributes to macrophage activation for killing *Leishmania* parasite (Mashayekhi Goyonlo et al., 2014). On the other hand, the inverse relation between IL-6 and IL-4 may explain the disease progression in patients from this group. IL-4 is known to be involved in the downregulation of Th1 cytokines, contributing to a non-healing response (Galgamuwa et al., 2019). Interestingly, IL-4 was also directly correlated with the parasite load. Kumar et al. (2009) obtained similar results connecting IL-4 and parasite burden but for primary CL patients. Nevertheless, there is a pattern where the parasite load associated with the upregulation of IL-4 leads to the inhibition of the protective immune response (Kébaïer et al., 2001; Kumar et al., 2009).

Importantly, our study presents limitations, such as the low number of recruited patients, limited recruitment sites, and low number of sequenced samples. Also, the fact that the UHB patients were recruited in a dermatologic unit, represents a bias of our study, and explain the more frequent active lesions observed in the UHB cohort. Still, the variety of clinical presentations and different species causing the disease confirm the complexity of the pathogenesis of TL showing that even patients with the same background develop different symptoms. We also acknowledge that several aspects of the host might influence cytokine levels, such as individual polymorphisms. Such gene polymorphisms can regulate the production and establishment of cytokines and determine patient susceptibility or resistance for TL and must be further investigated (Amorim et al., 2019; Shehadeh et al., 2019; Lera-Nonose et al., 2021). Despite possible individual differences, the canonical analysis revealed different clusters for each manifestation, which was evident mainly for the group of asymptomatic patients compared to the others.

Taken together, our work has demonstrated that patients with different clinical presentations of TL showed similar profiles of parasite load, antibody production, and cytokines, suggesting that these factors alone are not determinant for TL symptoms. This result also shows why it is not a good idea to look at these factors in isolation for understanding the progression of TL. However, when we observe how these factors are correlated to each other, it is possible to verify that these factors and especially the bifunctional cytokines, with roles in Th1 and Th2 responses, are fundamental in modulating the individual’s inflammatory response. The way these factors relate to each other can shed some light to explain the complex pathogenesis of TL (Volpedo et al., 2021).

The conduction of additional studies regarding the existing autochthonous *Leishmania* species combined with the verification of patient migration is imperative for a better determination of the *Leishmania* species distribution in Brazil, as well as the confirmation of the presence of *L. guyanensis* in the Federal District. The expansion of current knowledge regarding the influence of the host immune response in TL, gene polymorphisms, treatment failure, and parasite diversity are important for a better understanding of the disease pathogenesis, improving patient care.

## Data availability statement

The datasets presented in this study can be found in online repositories. The names of the repository/repositories and accession number(s) can be found in the article/**Supplementary Material**.

## Ethics statement

The studies involving human participants were reviewed and approved by Ethical Committee of Research at Faculty of Medicine/UnB (CAAE: 53157115.5.0000.5558/2016 and CAAE: 56709316.3.0000.5558/2015). The patients/participants provided their written informed consent to participate in this study.

## Author contributions

BCC and NN conceived and designed the experiments. CMG, EN, JO, and NN collected clinical samples. ACR, BC, and TV carried out the experiments. BD performed statistical analysis. BC, JLC, LH, MH, NN and BD analyzed the data. BC, JC and NN wrote the manuscript. All authors read and approved the final manuscript.

## Funding

This study was financed by Foundation for Advancement of Science (FAPDF - N° 193.000.965/2015), The National Research Council, Ministry of Science and Technology (CNPq/MCT) and The Agency for Training Human Resources, Ministry of Education (CAPES/ME), Brazil.

## Acknowledgments

We thank Dr. Tatiana Karla Borges for her expertise and assistance with flow cytometry experiments.

## Conflict of interest

The authors declare that the research was conducted in the absence of any commercial or financial relationships that could be construed as a potential conflict of interest.

## Publisher’s note

All claims expressed in this article are solely those of the authors and do not necessarily represent those of their affiliated organizations, or those of the publisher, the editors and the reviewers. Any product that may be evaluated in this article, or claim that may be made by its manufacturer, is not guaranteed or endorsed by the publisher.
