# Supplementary material for: Multiparametric analysis of host and parasite elements in new world tegumentary leishmaniasis
Source: Front Cell Infect Microbiol. 2022 Aug 9;12:956112. doi: 10.3389/fcimb.2022.956112 (PMC9395741; doi:10.3389/fcimb.2022.956112)
Supplement: Supplementary file 1 [file DataSheet_1.docx]

**SUPPLEMENTARY MATERIAL**


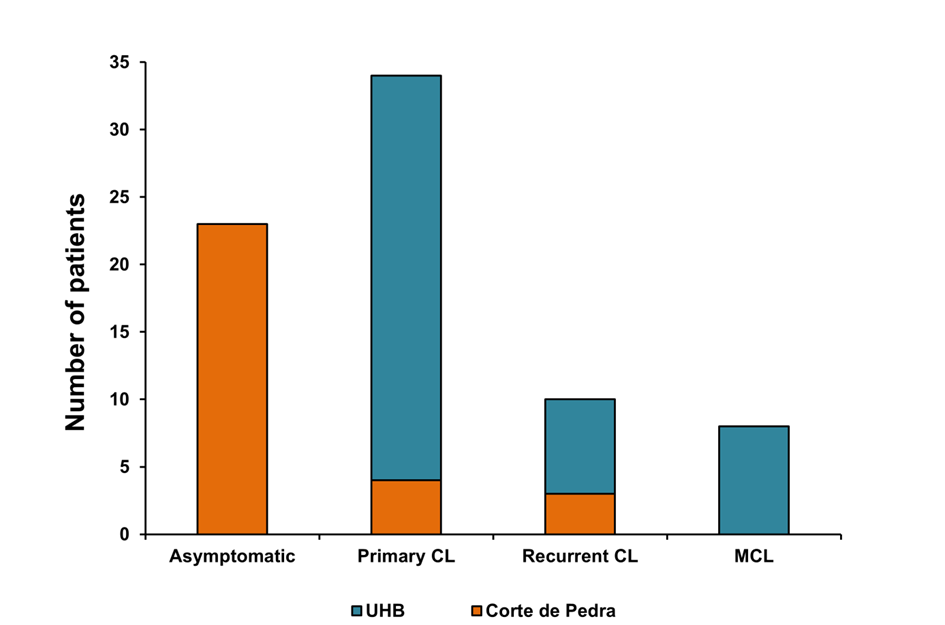


**Figure S1**- Classification of patients according to clinical manifestation and recruitment site. CL- Cutaneous leishmaniasis, MCL- Mucocutaneous leishmaniasis.

**Figure S2**- (A) Melting curve demonstrating the melt curve of *Leishmania* spp kDNA amplicons. (Black) Sample without DNA; (Green) Negative control, DNA from uninfected HEK cells; (Blue) Positive sample, DNA from TL patient; (Red) Positive control, DNA from *Leishmania braziliensis* culture; (B) Standard curve produced from different concentrations of *Leishmania braziliensis* DNA (R^2^=0.970, slope= - 3.228, efficiency=104%). The image sizes have been adjusted to fit the panel.

**Table S1-** *Leishmania* species identified by sequencing of ITS1 PCR products detected in studied individuals according BLASTn.


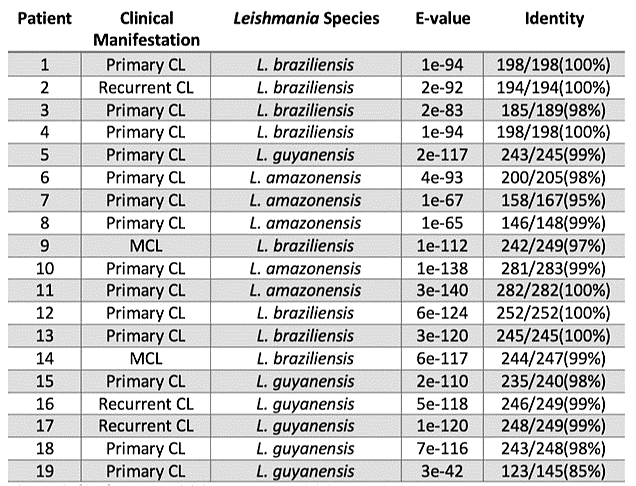


Legend: CL- Cutaneous leishmaniasis, MCL- Mucocutaneous leishmaniasis.


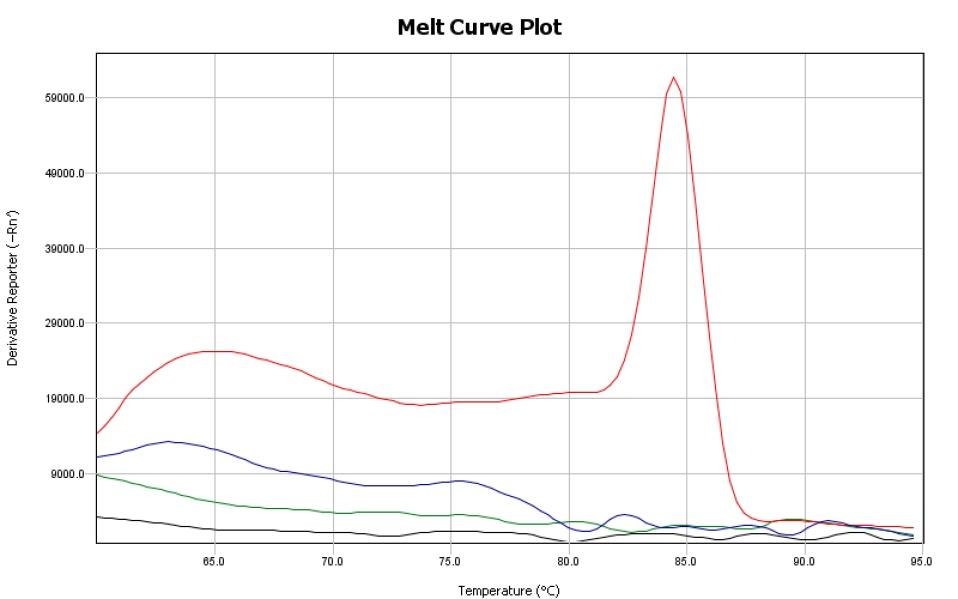


**Figure S3-** Melting curve of LRV1 amplicons. (Black) Sample without cDNA; (Blue) Negative control, cDNA from *Leishmania infantum*; (Green) Negative sample, cDNA from biopsy of TL patient; (Red) Positive control, cDNA from *Leishmania guyanensis* positive for LRV1.

**Table S2-** Cytokines concentration by clinical manifestations. Statistical significance was calculated using Kruskal-Wallis test.

| Cytokine |  | Clinical Manifestation | | | | | | | | | | | | | | |  | P value |  | Hedges'g value (range) |
| --- | --- | --- | --- | --- | --- | --- | --- | --- | --- | --- | --- | --- | --- | --- | --- | --- | --- | --- | --- | --- |
|  |  | Asymptomatic | | |  | MCL | | |  | Primary CL | | |  | Recurrent CL | | |  |  |  |  |
|  |  | Mean |  | SD |  | Mean |  | SD |  | Mean |  | SD |  | Mean |  | SD |  |  |  |  |
| IFN-y |  | 0.72 |  | 2.85 |  | 18.14 |  | 44.74 |  | 0.21 |  | 0.49 |  | 0.66 |  | 1.36 |  | ns |  | 0.30 - 0.61 |
| IL-2 |  | 4.4 |  | 10.41 |  | 1.51 |  | 1.53 |  | 2.95 |  | 2.17 |  | 13.34 |  | 29.11 |  | ns |  | 0.26 - 0.36 |
| IL4 |  | 0.62 |  | 1.95 |  | 0.32 |  | 0.52 |  | 0.49 |  | 1.24 |  | 10.43 |  | 27.75 |  | ns |  | 0.08 - 0.52 |
| IL-6 |  | 3.78 |  | 3.95 |  | 3.45 |  | 1.67 |  | 6.17 |  | 7.45 |  | 6.06 |  | 6.37 |  | ns |  | 0.10 - 0.44 |
| IL-10 |  | 0.22 |  | 0.54 |  | 0.41 |  | 0.5 |  | 0.28 |  | 0.67 |  | 0.61 |  | 1.14 |  | ns |  | 0.10 - 0.45 |
| IL-17A |  | 0.81 |  | 3.14 |  | 3.73 |  | 4.93 |  | 3.07 |  | 4.34 |  | 2.69 |  | 6.22 |  | ns |  | 0.39 - 0.77 |
| IgG |  | 1.04 |  | 1.17 |  | 1.96 |  | 1.26 |  | 1.09 |  | 1.2 |  | 1.47 |  | 1.5 |  | ns |  | 0.32 - 0.77 |
| TNF |  | 2.65 |  | 9.48 |  | 0.26 |  | 0.66 |  | 0.52 |  | 0.91 |  | 8.77 |  | 24.89 |  | ns |  | 0.33 - 0.45 |

Legend: **SD** – Standard Deviation, **ns**- not significant, **IL-2**- interleukin 2, **IL-4**- interleukin 4, **IL-6**-interleukin 6, **IL-10**- interleukin 10, **IL-17A**- interleukin 17A, **IFN-y**- interferon y, **TNF**- tumor necrosis factor. **CL**- Cutaneous leishmaniasis, **MCL**- Mucocutaneous leishmaniasis.


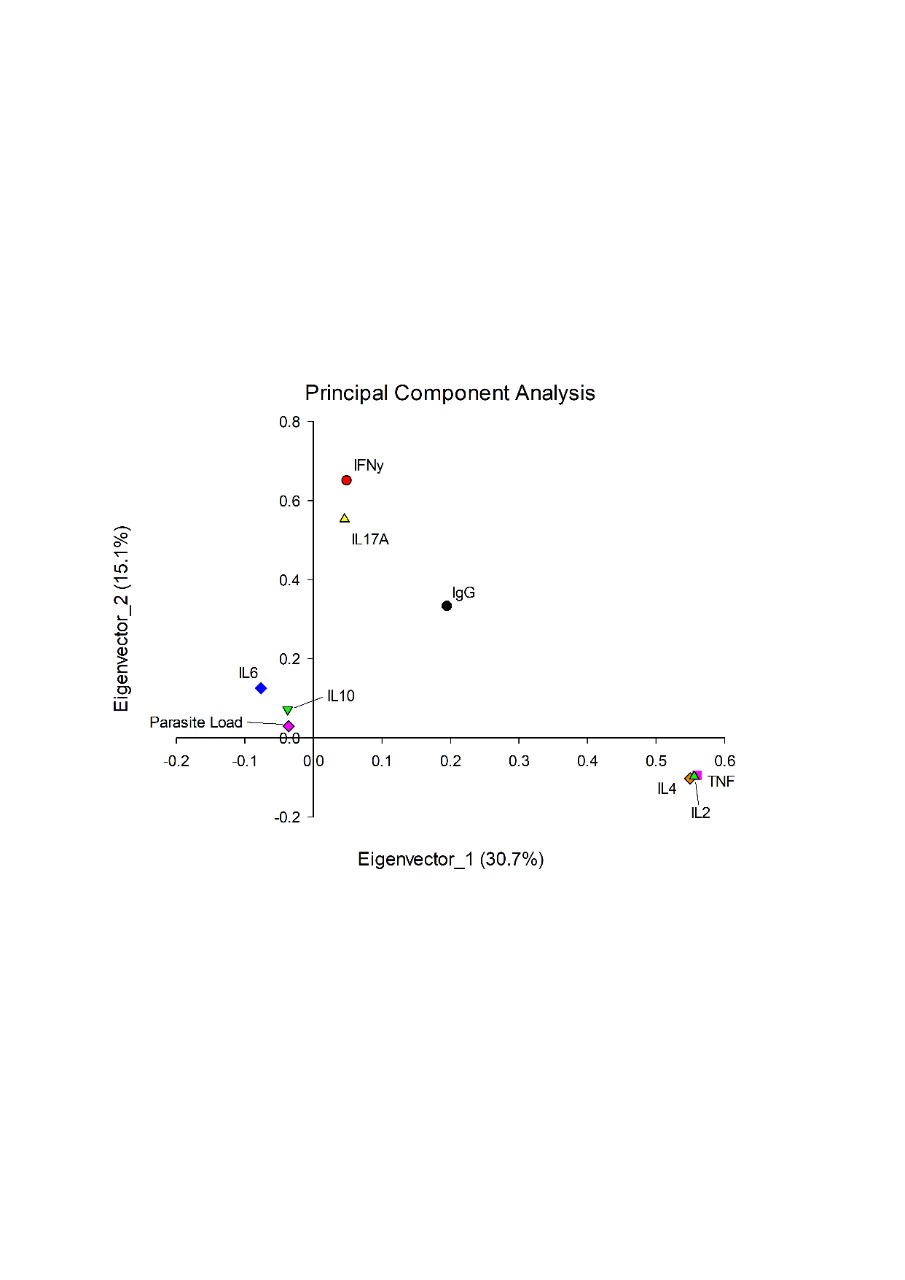


**Figure S4-** Principal component analysis. Eigenvectors of evaluated parameters of Tegumentary leishmaniasis pathogenesis. The value in parentheses represents the variation that was explained by each eigenvector. Elements of the same quadrant behave similarly. IL: interleukin. IFN: interferon γ. TNF: tumor necrosis factor.
